# Long‐Range Forces in Rock‐Salt‐Type Tellurides and How they Mirror the Underlying Chemical Bonding

**DOI:** 10.1002/adma.202100163

**Published:** 2021-07-29

**Authors:** Jan Hempelmann, Peter C. Müller, Philipp M. Konze, Ralf P. Stoffel, Simon Steinberg, Richard Dronskowski

**Affiliations:** ^1^ Institute of Inorganic Chemistry RWTH Aachen University D‐52056 Aachen Germany; ^2^ Jülich‐Aachen Research Alliance (JARA‐CSD) RWTH Aachen University D‐52056 Aachen Germany; ^3^ Hoffmann Institute of Advanced Materials Shenzhen Polytechnic 7098 Liuxian Blvd, Nanshan District Shenzhen 518055 China

**Keywords:** chemical‐bonding analysis, hyperbonding, incipient metals, metavalent bonding, phase‐change materials, projected force constants

## Abstract

Chemical bonding in main‐group IV chalcogenides is an intensely discussed topic, easily understandable because of their remarkable physical properties that predestine these solid‐state materials for their widespread use in, for instance, thermoelectrics and phase‐change memory applications. The atomistic origin of their unusual property portfolio remains somewhat unclear, however, even though different and sometimes conflicting chemical‐bonding concepts have been introduced in the recent years. Here, it is proposed that projecting phononic force‐constant tensors for pairs of atoms along differing directions and ranges provide a suitable and quantitative descriptor of the bonding nature for these materials. In combination with orbital‐based quantitative measures of covalency such as crystal orbital Hamilton populations (COHP), it is concluded that the well‐established many‐center and even *n*‐center bonding is an appropriate picture of the underlying quantum‐chemical bonding mechanism, supporting the recent proposal of hyperbonded phase‐change materials.

## Graphical abstract

Examining the chemical bonding in rock‐salt‐type IV–VI functional materials by projected force constants as derived from ab initio thermochemistry yields unusually strong long‐range forces along the cubic lattice vectors, impossible to account for by either two‐center covalent or ionic bonding. The full analysis, including orbital‐based bonding descriptors, points toward hyperbonding, thereby explaining certain unique properties of this material class.

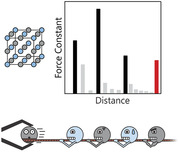

## 1 Introduction

In the quest for new materials addressing future challenges, solid‐state chalcogenides have gained enormous interest due to their remarkable chemical and physical properties, thereby placing them at the forefront of basic research and technology.^[^ ^1^ ^]^ Among the chalcogenides, main‐group IV–VI materials such as GeTe, PbTe, or SnSe are of particular interest since they play a decisive role in numerous applications.^[^ ^2^ ^]^ For instance, PbTe and SnSe exhibit unusually high *ZT* values making them ideal materials for thermoelectric energy conversion.^[^ ^3^ ^]^ On the other side, data‐storage phase‐change materials like GeTe are capable of fast resistive or optical switching between amorphous and crystalline states while simultaneously exhibiting a stark contrast in electrical or optical properties.^[^ ^4^, ^5^ ^]^ Despite widespread use, the theoretical understanding of these unusual characteristics is still somewhat incomplete although some chemical features are obvious: for example, all three aforementioned compounds have a valence‐electron count of 10 and thus violate the 8 − *N* rule. This results in a significant amount of metal–nonmetal antibonding interactions close to the Fermi level,^[^ ^6^, ^7^ ^]^ which nature rectifies, at least in part, through the formation of vacancies, by structural distortions, and even by the introduction of new homopolar bonds.^[^ ^6^, ^8^, ^9^ ^]^

Among the IV–VI materials, there is also a broad abundance of rock‐salt‐type and similar structures. Even in the related pseudo‐binary GeTe–Sb_2_Te_3_ (“GST”) system, phase‐change materials such as Ge_2_Sb_2_Te_5_ or Ge_1_Sb_2_Te_4_ adopt a metastable [NaCl]‐type phase, which may eventually recrystallize into a layered and stable hexagonal phase.^[^ ^10^ ^]^ The prevalence of the rock‐salt type looks surprising at first, as it is typically encountered for materials comprising predominantly ionic bonding, while more covalent materials (say, ZnS) prefer structure types with lower coordination numbers.^[^ ^11^ ^]^ And yet, the electronegativity differences in these functional chalcogenides are usually small, so large ionic charges are not found.^[^ ^12^, ^13^ ^]^ The amorphous phase maintains some of the structural motifs of the crystalline phase, such as axial units comprising of three collinear atoms, while the rest of the coordination polyhedron is distorted. The additional formation of homopolar bonds lowers the coordination number to be closer to the value predicted by the 8 – *N* rule.^[^ ^14^ ^]^

The combination of the aforementioned structural features and remarkable physical properties suggests unique electronic structures for these chalcogenides. That being said, it is hardly surprising that there have been several attempts to both categorize and explain the phenomena. The very first model tried to apply the “resonant‐bonding” concept,^[^ ^15^ ^]^ known from organic chemistry and the benzene molecule, to deal with the materials’ electron excess, and a given structure was considered an intermediate (“resonating”, valence‐bond language) state between two distinct and purely covalent lattices. Over the past years, this concept has been revised and also recoined under the term “metavalent” bonding, a novel and essentially unique bonding mechanism between metallic and covalent bonding, solely encountered for the family of incipient metals.^[^ ^16^ ^]^ At the same time, “metavalent” bonding was tied to a portfolio of distinctive, experimentally accessible materials properties and certain density‐based theoretical descriptors associated with the ionic and covalent character in the materials’ chemical bonding.^[^ ^17^, ^18^ ^]^ For instance, the experimentally accessible signposts included laser‐induced atom probe tomography (APT) data indicating a specific bond‐breaking behavior for incipient metals.^[^ ^19^ ^]^ However, all of these quantities listed in the portfolio do not provide an adequate picture of the bonding nature in an orbital‐based language. More recently,^[^ ^20^ ^]^ a “hyperbonding” mechanism was introduced to rationalize several of the aforementioned properties and peculiarities found in the IV–VI family of phase‐change materials. In connection with this model, it was proposed that the electron surplus resulting from violating the 8 – *N* rule manifests in 3‐center 4‐electron bonds in chalcogenide phase‐change materials, leading to 3‐atom linear structural motifs in both crystalline and amorphous phases. It goes without saying that terms such as 3‐center 4‐electron interactions eventually allude to wave functions from quantum chemistry, in particular molecular‐orbital theory,^[^ ^21^ ^]^ breaking the inherent limits of density‐based schemes.

Herein, we present another, yet related approach based on ab initio phonons and orbital‐based bonding indicators to shed light on the peculiar bonding nature of IV–VI materials. Ab initio phonons were chosen because they allow revealing the bonding situation, accurately so, by mirroring the interatomic forces. Given accurate forces and phonons,^[^ ^16^, ^22^ ^]^ theoretical thermodynamic parameters may correctly quantify some of the materials’ properties at finite temperature. In addition, we here utilize the recently established bond‐projected force constants (BPFCs),^[^ ^23^ ^]^ derived from the force tensors of phononic calculations, which are essential if one wants to break down the entire phononic analysis to pairs of atoms, similar to what is usually done in the case of the electronic structure. For the latter, we simultaneously evaluate the respective crystal orbital Hamilton populations (COHP) whose energy integrals (ICOHP) serve as a measure of covalent bond strength. The resulting analysis therefore incorporates both lattice dynamics, as well as orbital‐based results. Anticipating things a bit, our analyses encourage a concept of multicenter bonding to which the aforementioned hyperbonding also belongs to.

## 2 Computational Methodology

Density‐functional theory (DFT) was used to first optimize the investigated structures and, second, based on those structures to calculate the atomic dislocations needed for simulating the phonons with PHONOPY.^[^ ^24a^ ^]^ Herein, all DFT‐based computations were conducted utilizing the projector augmented wave^[^ ^25^ ^]^ method as implemented in the Vienna Ab initio Simulation Package^[^ ^26^ ^]^ (VASP). PBEsol^[^ ^27^ ^]^ functionals were used to fully optimize the structures before constructing supercells and dislocating single atomic positions.

Generally, the projected force constants Φ_p_ are calculated from harmonic force constants, generated as Cartesian force constant matrices for each atom pair κ, κ′. As such, they can easily be projected along their respective interatomic vectors, as outlined in previous work^[^ ^23^ ^]^

(1) $$\begin{array}{c} \Phi_{P}(\kappa \kappa^{\prime })=|(\begin{array}{ccc} \Phi_{\kappa \kappa^{\prime }}^{xx} & \Phi_{\kappa \kappa^{\prime }}^{xy} & \Phi_{\kappa \kappa^{\prime }}^{xz}\\ \Phi_{\kappa \kappa^{\prime }}^{yx} & \Phi_{\kappa \kappa^{\prime }}^{yy} & \Phi_{\kappa \kappa^{\prime }}^{yz}\\ \Phi_{\kappa \kappa^{\prime }}^{zx} & \Phi_{\kappa \kappa^{\prime }}^{zy} & \Phi_{\kappa \kappa^{\prime }}^{zz} \end{array})(\frac{r(\kappa^{\prime })-r(\kappa )}{\left|r(\kappa^{\prime })-r(\kappa )\right|})| \end{array}$$

It makes intuitive sense that projected force constants opposite to each other along the diagonal, Φ_p_(*κκ*′) and Φ_p_(κ′κ), should be identical, as they describe a single pair of atoms. PHONOPY calculates them, however, by moving one of the atoms and, as such, the structural environment of the displacement may be dissimilar for unequal crystallographic positions. Consequently, in most cases Φ_p_(*κκ*′) ≠ Φ_p_(κ′κ). This is not surprising as the underlying matrices’ symmetric relations are known. If a crystal structure is dynamically stable, matrix elements are symmetric following the equation^[^ ^24b^ ^]^

(2) $$\Phi_{\kappa \kappa^{\prime }}^{\alpha \beta }=\frac{\partial^{2}U}{\partial u_{\kappa }^{\alpha }\partial u_{\kappa^{\prime }}^{\beta }}=\frac{\partial^{2}U}{\partial u_{\kappa^{\prime }}^{\beta }\partial u_{\kappa }^{\alpha }}=\Phi_{\kappa^{\prime }\kappa }^{\beta \alpha }$$

for a potential *U*, the dislocation *u* and the Cartesian indices α, β. This symmetry implies that a projected force constant value may be different for an atom pair κ,  κ′, depending on which of the two atoms is dislocated from its equilibrium position. The atomic environments of κ and κ′ both contribute to their overall interaction and are averaged into a single complete projected force constant.

**Figure 1:**
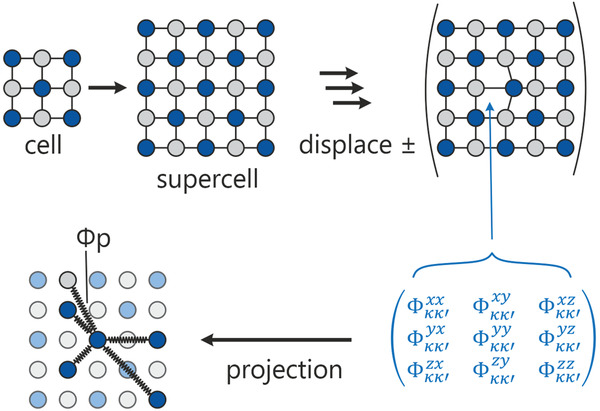
Schematic overview of the force‐constant projection procedure. A supercell is generated from a relaxed unit cell containing *x* atoms, then individual atoms are displaced in ± directions along all three spatial directions. By doing so, *x* ^2^ Cartesian force‐constant tensors are received through harmonic approximation during the phonon simulation. These tensors are then projected along interatomic vectors to generate individual force constants for each given atomic pair within the cell.

This very method provides us with one force‐constant value for any pair of atoms within the calculated supercell. For the calculation, the projected force constants require the presence of a full coordination polyhedron within the supercell, and they are only independent of its size for interatomic distances smaller or equal than half the supercell, due to translation symmetry (**Figure** **1**).

The projected force constants were then related to the crystal orbital Hamilton populations (COHP),^[^ ^28^, ^29^ ^]^ which are obtained by weighting the off‐diagonal entries of a given density‐of‐states matrix with the respective elements of the corresponding Hamilton matrix. In doing so, it is straightforward to identify bonding, non‐bonding, and antibonding interactions in a given material directly from the wave function. Because COHP analysis requires a local orbital basis set whose nature is in stark contrast to the delocalized plane waves (see above), projected COHPs were analytically projected from the latter by means of the Local‐Orbital Basis Suite Towards Electronic‐Structure Reconstruction (LOBSTER) code.^[^ ^28^, ^30^ ^]^ Because the entire electronic structure is reconstructed, all other local measures automatically fall off from the calculations such as orbital and atomic gross populations as well as charges from Mulliken and Löwdin^[^ ^31^ ^]^ analyses.

## 3 Results and Discussion

### 3.1 Unique Long‐Range Interactions in GeTe

**Figure 2:**
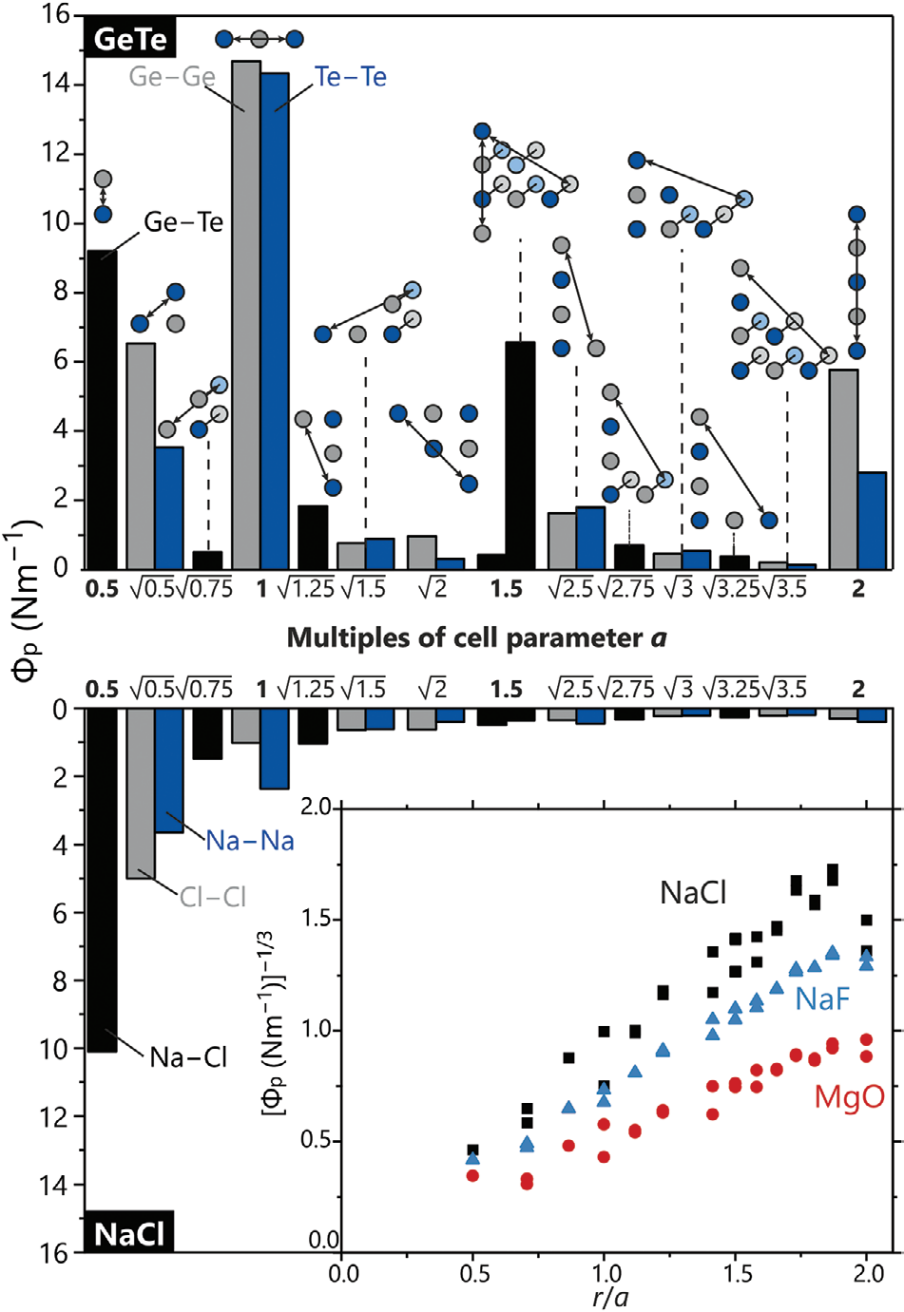
Projected force constants for various pairs of atoms within rock‐salt‐type GeTe (top) and NaCl (bottom) up to a distance of two lattice parameters *a*, with heteropolar forces in black, homopolar in atomic colors. While GeTe exhibits numerous force‐constant maxima when atoms align along interatomic vectors at distances highlighted in bold, the NaCl‐derived values steadily decrease following an isotropic relationship with distance. Inlay: “Linearized” force constants of NaCl according to Badger's rule.^[^ ^33^ ^]^ Other ionic rock‐salt‐type structures show the same behavior.

To demonstrate the unusual lattice dynamics observed for IV–VI materials by means of projected force constants, we first chose a model system crystallizing with a common structure model and, at the same time, exhibiting the aforementioned remarkable properties. Because rock‐salt‐type germanium telluride, which is a prototypical^[^ ^5^ ^]^ phase‐change material as well as a promising^[^ ^32^ ^]^ compound for mid‐temperature thermoelectrics, fulfills these preconditions, this very telluride was considered as an ideal model system. **Figure** **2** compares the force constants of the cubic (β‐type) GeTe in the upper frame with those of real NaCl in the lower frame, for distances up to two lattice parameters. It is obvious that GeTe exhibits unusual force‐constant maxima at distances well beyond the ranges of conventional covalent bonding. These extrema appear whenever additional atoms are stacked along the connecting vector between the two examined ones. This is the case for the force constants projected along the lattice parameter *a* at distances of 1*a*, 1.5*a*, and even 2*a*, and the outcome suggests a strong directional dependence of forces becoming especially apparent for Φ_p_ values at 1.5*a* (bold in Figure 2). Here, the rock‐salt type allows for two interatomic vectors with the same length, one parallel to the lattice vectors, the other one cutting diagonally through the cell (as depicted in the pictograms in Figure 2). Hence, the lattice‐aligned force constant is very large, reaching two thirds of the nearest‐neighbor force constant, despite the considerable increase in distance between the two atoms. In stark contrast, the diagonal and unaligned constant only amounts to a fraction of the lattice‐aligned one. It is worth mentioning that these interactions appear for Ge–Ge, Ge–Te and Te–Te atomic pairs. Ge–Ge and Te–Te pairs exhibit very similar force constants for 1*a*, while the Te–Te value falls to half of that of the Ge–Ge one for 2*a* suggesting that these homoatomic interactions behave differently at wider distances.

This outcome agrees well with previous findings^[^ ^22^ ^]^ providing a link between “resonant” bonding and low lattice thermal conductivity. Namely, unusually high values in the force constant matrices were detected, so a single‐value descriptor was generated by calculating the traces of these matrices and normalizing them using the traces of their respective self‐interaction tensors. Unfortunately, every method based on the trace of a given force constant tensor will always depend on the unit cell chosen. As such, it will deliver drastically different values for the investigated [NaCl]‐type structures depending on whether the conventional or the primitive cell is used. Projection of the force constants, however, solves this problem and does not require any normalization as it provides an approximation of the true force constants in the compound. In contrast to the trace‐based method, the projected force constants show better resolution of the long‐range interactions across all distances, especially at 2*a*.

It is tempting to interpret the GeTe force‐maximum irregularities to result from numerical instabilities of the phonon calculations, but such objection can be ruled out since ionic rock‐salt‐type compounds including NaCl itself do not exhibit this behavior at all. Instead, as depicted in the lower frame of Figure 2, all NaCl force constants of the same type steadily decrease with growing interatomic distance. For illustration, let us focus on the projected force constants for the 1.5*a* distance showing a massive directional preference of certain forces in GeTe, but nothing like that is observed in NaCl where there is barely any difference at all. In fact, the lattice‐aligned force constant is slightly smaller than the non‐aligned one.

While the strong anisotropy in GeTe is difficult to comprehend and model, the general decline of interaction strength with increasing interatomic distance for a material following the 8 – *N* rule such as NaCl is strikingly regular. Indeed, it can be linearized by plotting the inverse third cubic root 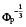 against the interatomic distance (inlay in Figure 2), which amounts to the same proportionality as described in Badger's iconic rule^[^ ^33^ ^]^ for diatomic molecules with two elements *i* and *j*

(3) $$k^{-\frac{1}{3}}\;\;=\;\;a_{ij}\left(D_{0}-b_{ij}\right)$$

where *k* denotes the interatomic force constant, *D*_0_ is the equilibrium distance, and *a_ij_* and *b_ij_* are element‐specific coefficients. It was also shown, by Waser and Pauling, that the rule nicely holds for typical solids when regarding the nearest‐neighbor bond force constants.^[^ ^34^ ^]^ This correlation was commonly used to describe trends within related compounds of elements within the same period. As such, it is interesting that it also accurately describes the force constants of many different bonds, all within the same compound and far beyond the first coordination sphere and typical covalent bond lengths.

The uniform decrease found for NaCl essentially points to the lack of any directional preference of forces within the crystal structure, hence the behavior agrees well with the omnidirectional nature of ionic bonding and can indeed be confirmed for other ionic [NaCl]‐type salts such as NaF and MgO, also shown in the inlay of Figure 2.

**Figure 3:**
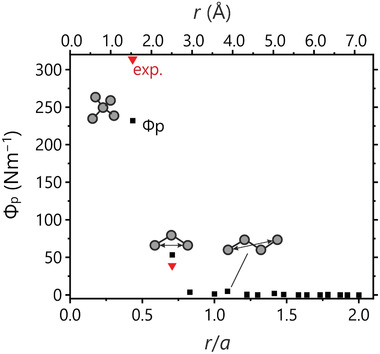
Projected force constants for diamond plotted against the bond length, showing that the force constants decrease sharply upon increasing interatomic distance. Due to the greater number of non‐equivalent interatomic distances, only interatomic vectors that divert from the overall trend have been described with a pictogram.

For chemists, the difference in projected force constants for two compounds such as GeTe and NaCl is hardly surprising, simply because the latter is well known for its ionicity while the former barely exhibit any charges at all,^[^ ^12^ ^]^ so covalency should be at play. This circumstance is easily recognized from the wave function‐computed Mulliken (±0.00) and Löwdin (±0.18) charges, and together with the bond trajectory shown by the projected force constants this indirectly confirms some sort of covalent interaction as the dominant bonding mechanism in GeTe. On the other hand, one would intuitively expect the force constants of a covalent material to also decrease sharply after the first coordination sphere as the bonds are directional and the interactions are rather short‐ranged. Indeed, we can confirm this expectation from the example of diamond, the covalent archetype. **Figure** **3** depicts its force constants as a function of the interatomic distance, making the difference to the ionic compounds and GeTe immediately apparent. The force constants decrease sharply with increasing C—C distance. While the nearest‐neighbor value amounts to 230 N m^−1^, the second‐nearest value is considerably smaller at 50 N m^−1^, but still large compared to those at longer ranges which are close to zero. Experimentally, the spectroscopy‐derived force constants arrive at 314 and 39 N m^−1^ for these pairs, respectively.^[^ ^35^ ^]^ We explain the deviation from experiment by the use of the harmonic approximation and the absence of temperature dependence for the projected force constants. However, the semi‐quantitative course of the theory is correct, in particular the significant size of the second‐sphere force constant. Another example of high connectivity but with a non‐(8 − *N*) electron count would be given by tungsten carbide, WC, and its 6/6 connected trigonal prisms. Indeed, preliminary calculations likewise indicate the existence of long‐ranged interactions in WC coinciding with the *c* lattice parameter, and they will be further studied in future work.

**Figure 4:**
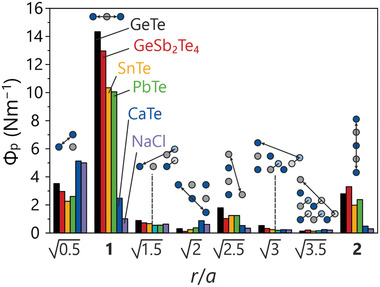
Comparison of VI–VI projected force constants in Te–Te‐type tellurides, as well as CaTe and NaCl for reasons of comparison. The unusual long‐range force constants are present for all IV–VI compounds while neither CaTe nor NaCl show comparable effects.

When compared to either NaCl or diamond, cubic germanium telluride's unusual force constants can neither be accounted for by ionic nor by conventional (i.e., two‐center) covalent bonding. While the strong directional anisotropy is indicative of covalency, the fourth coordination sphere with interatomic distances of more than 6 Å exhibiting the largest force constants is incompatible with a two‐center covalent bonding mechanism. Likewise, the various force‐constant entries in **Figure** **4** evidence that GeTe shares this quality with other rock‐salt IV–VI type compounds like SnTe and PbTe, but rock‐salt‐type tellurides like CaTe which follow the 8 – *N* rule do not exhibit this behavior at all but resemble NaCl, the iconic 8 – *N* representative.

Hence, it seems reasonable to assume that these long‐range interactions are indeed a common property of rock‐salt IV–VI materials and, as such, are likely to be connected to (or even causing) their special properties.

### 3.2 Vacancy Effects on Force Constants

Besides the common occurrence of rock‐salt‐derived structure types, IV–VI materials also often exhibit a significant amount of vacancies in the main‐group IV sublattice, thereby resulting in a lower valence‐electron count which translates into (partially) depopulated antibonding levels.^[^ ^6^, ^8^ ^]^ Since all previous calculations assumed full atomic occupancy, it is prudent to consider the impact of missing atoms. To do so, projected force constants were calculated for 2 × 2 × 2 supercells (then doubled to 4 × 4 × 4 for the phononic calculations) with randomly distributed vacancy positions. For a selection of tellurides, a comparison of the projected force constant averaged over all Te–M–Te units to the value for a vacancy‐mediated Te–□–Te unit reveals the effect of those vacant positions. If the bridging Ge atom is removed, the unusually strong interaction disappears. This reinforces the notion that the electronic excess injected into the interaction by divalent Ge is responsible for its unusual strength. Calculations for [NaCl]‐type GeSb_2_Te_4_ (“GST124”) show that even higher vacancy concentrations do not inhibit the formation of these long‐range interactions (see again Figure 4).

**Figure 5:**
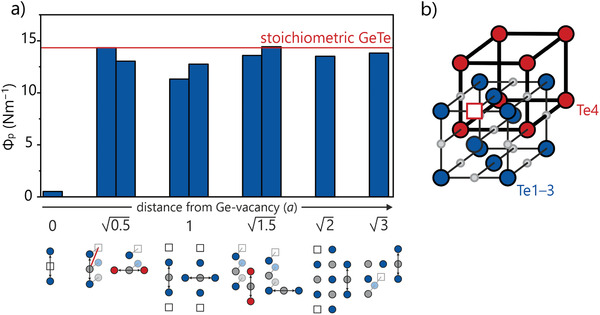
a) Graphical analysis of Te–Ge–Te interactions in Ge_31_Te_32_ in relation to their respective spacing from the Ge vacancy. The distance is measured from the central Ge atom and given in units of the lattice parameter *a*. As the influence of the vacancy is mostly local, those interactions occurring at larger distances approach the value of stoichiometric GeTe. Notably, Te–Ge–Te interactions at the same distance from the vacancy may shift differently. b) The reason is found, at least in part, in the different natures of the tellurium sublattices, three of which include atoms that are in immediate contact with the vacancy (blue, Te1–3), while one does not (red, Te4). They correspond to the four Te positions in stoichiometric GeTe.

**Figure** **5** elaborates on this by resolving the local effect of a single Ge vacancy in Ge_31_Te_32_ in its chemical environment. Clearly, the force constant is significantly attenuated only if evaluated in the vacancy's immediate vicinity. Otherwise, the projected force constants approach the value of the vacancy‐free structure upon increasing the distance to the vacancy. This suggests that the statistical appearance of vacancies does not significantly impact the bonding situation of the compound, so it is a local phenomenon, not caused by the underlying electron‐density count. Remarkably, some Te–Ge–Te force constants may shift somewhat differently depending on the Te atoms involved. For the case of the interaction occurring at a distance of 1*a* ( = a single lattice parameter) between the vacancy and the center of the Te–Ge–Te bond, this difference is easily explained by one of the atomic chains being interrupted frequently, while the other remains intact. At distances of $\sqrt{0.5}a$ and $\sqrt{1.5}a$ the situation differs: upon removal of a Ge atom, the formerly symmetry‐equivalent Te atoms form four simple cubic sublattices (see Figure 5b) and three of those Te lattices contain the Te–□–Te units showing larger force constants at these distances than the remaining Te lattice. This may be explained by the additional electrons from the Te–□–Te unit being available for long‐range interactions along these sublattices. While the overall effect of the vacancy on long‐range forces remains a weakening one, individual interactions may be barely affected or even (slightly) strengthened.

### 3.3 Displacements Shape Bonding

Let us reiterate that the projected force constants result from DFT phonon calculations where atoms are dislocated from their equilibrium positions in supercells. To shed more light on the unusual nature of these force constants, we also conducted bonding analyses based on projected COHPs for such perturbed structures. To do so, a single atom (Ge or Te) sitting in a 3 × 3 × 3 supercell was shifted by 0.2 Å, followed by a comparison of the resulting projected ICOHP values and Löwdin charges. While the projected COHP is particularly suited for quantifying short‐range covalent interactions, we will see that long‐range interactions as resolved by projected force constants still show up from COHP information beyond the nearest neighbor.

**Figure 6:**
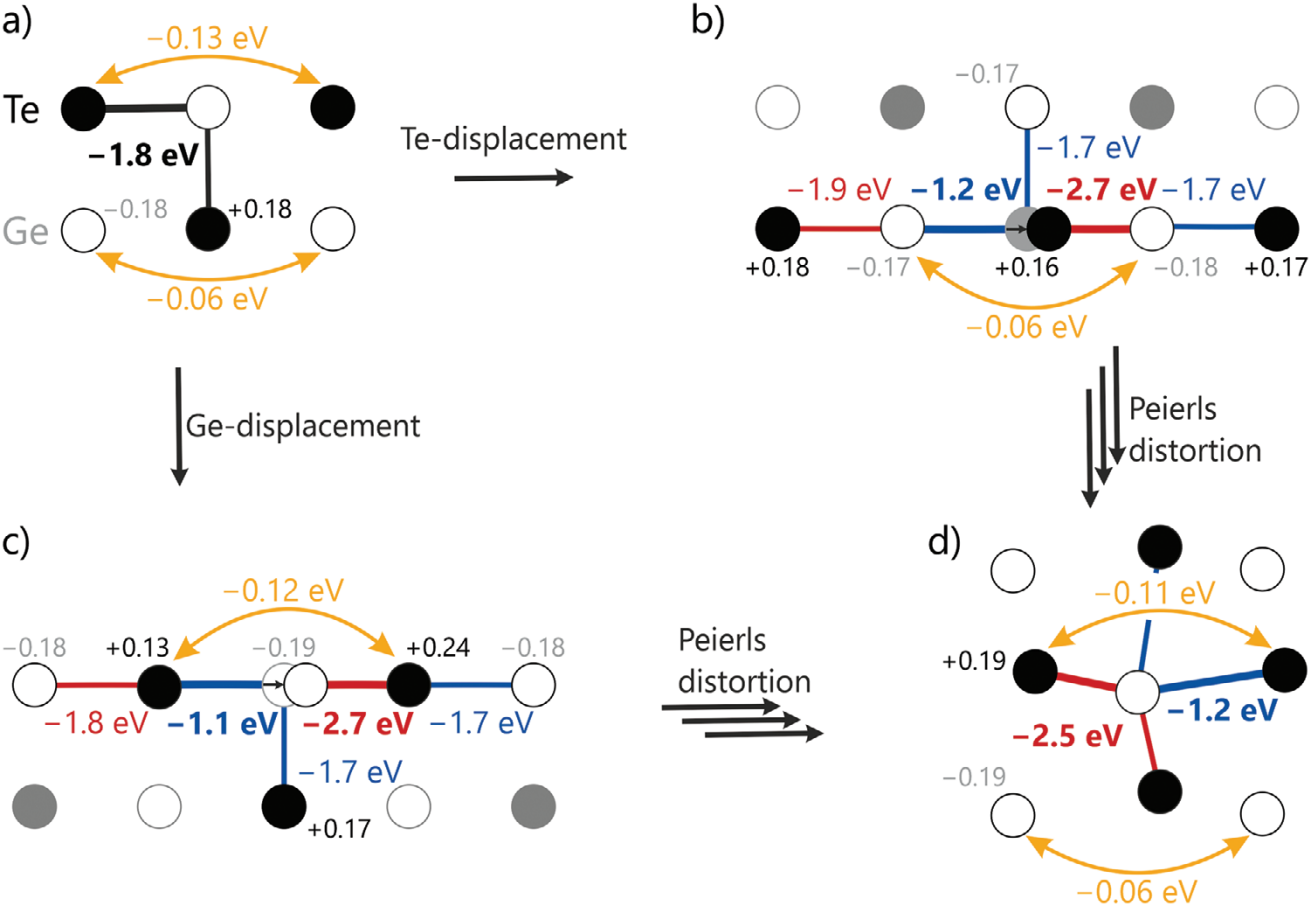
Effects of atomic displacements by 0.2 Å on bonding descriptors and charges in GeTe. a) Non‐perturbed cubic GeTe. b,c) Tellurium and germanium displacements respectively. d) α‐GeTe that is the result of a Peierls distortion, creating a shorter and a longer bond length. Projected ICOHP and Löwdin charges for relevant atoms and interactions are depicted.

In its unperturbed structure, GeTe is characterized by a strong nearest‐neighbor Ge–Te bond with an ICOHP of −1.8 eV (**Figure** **6**a) and very low Löwdin charges of ±0.18. In addition to that, there are much smaller but significant homoatomic orbital interactions, along a Te–Ge–Te unit (−0.13 eV) and involving a Ge−Te−Ge fragment which comes out even smaller (−0.06 eV). This clearly suggests a significant role of germanium in mediating the Te−Te interaction, not at all unexpected as germanium is the main‐group IV element carrying the two excess electrons. As such, one might argue that dislocating a Ge atom should have a stronger impact on these weaker interactions than moving Te. Indeed, this can be observed in Figure 6c, where the germanium displacement slightly weakens the Te−Te interaction (from −0.13 to −0.12 eV), while the analogous tellurium shift (Figure 6b) does not change the Ge−Ge one (a constant −0.06 eV). The Ge shift also induces a stronger change in the atomic charges inside the new Te−Ge−Te unit (+0.13/−0.19/+0.24), in turn suggesting a stronger disturbance of the structure for a Ge displacement. The increased ICOHP value of the dislocated 3‐atom units is best explained by the shorter distance between them, allowing for better orbital overlap.

Upon either Ge or Te displacement, the nearest‐neighbor ICOHP increases from   −1.8 to −2.7 eV along the displacement direction but decreases in all others, with the longer parallel interaction affected significantly more than the perpendicular one. Most remarkably, the nearest‐neighbor ICOHP values are alternatingly moving up and down in size, suggesting an incentive for dimerization along this chain. This makes such a dislocation a potential starting point for phase change. Corresponding shifts in the crystal structure lead to a Peierls distortion of β‐GeTe, which results in the formation of α‐GeTe (as depicted in Figure 6d). From the displacement follows a slight misalignment of the orbitals oriented perpendicular to the displacement direction, preventing full overlap. In turn, a small π‐interaction is formed with the nearest‐neighbor, toward which the atom was shifted. Preferential bonding with some neighboring atoms at the cost of interaction with others may be understood as a fractional lowering of the coordination number. Consequently, it is closer to the coordination number expected by the octet rule.

### 3.4 Collapsing the Rock‐Salt Structure

Let us reiterate the transformation between β‐GeTe and α‐GeTe. While the former is the most significant phase for switching, the latter low‐temperature hexagonal (rhombohedral) form is the result of a three‐dimensional Peierls distortion: six identical nearest‐neighbor Ge—Te bonds split into three longer “interlayer” and three shorter “intralayer” ones, so the coordination number lowers and gets closer to the value expected by the 8 – *N* rule. This bond‐length separation resembles the dimerization incentive observed for the ICOHP of the perturbed β‐GeTe structure, as α‐GeTe is formed through Peierls distortion.

**Figure 7:**
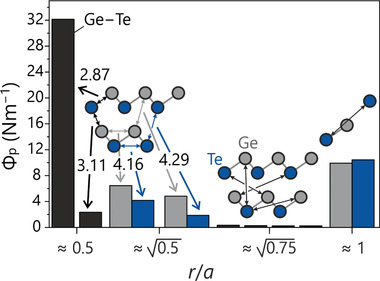
Projected force constants for α‐GeTe and atom–atom distances in relation to the pseudo‐cubic lattice parameter *a*. The apparent symmetry lowering compared to β‐GeTe leads to several different distances, the most significant given in Ångstroms. Note how the nearest‐neighbor interaction splits into one very strong and one very weak one.

The change from the ideal cubic case has a significant impact on the projected force constants, as shown in **Figure** **7**. For the three shorter bonds, the nearest‐neighbor interaction force is much stronger (about 32 N m^−1^) than in the cubic case, while the force constants for the three longer bonds are comparatively small (about 2 N m^−1^). Considering the layered structure of α‐GeTe, these results are in line with chemical intuition.

While being slightly weaker than in β‐GeTe, the long‐range 1*a* force is still present in α‐GeTe, despite near alignment with the two unequal, closer Ge−Te interactions, therefore connecting two different layers. Hence, the long‐range interactions that seem to be characteristic for the [NaCl] phase‐change materials, and for GeTe in particular, are still somehow present in the distorted low‐temperature phase; yet, short‐ranged effects appear to be dominant at these temperatures, distorting the structure. Starting from these remaining long‐range interactions, α‐GeTe may also recrystallize into the metastable cubic structure. Accordingly, the projected force constants beyond the nearest‐neighbor sphere in both α‐GeTe and β‐GeTe indicate cooperating long and short‐range effects; however, in the case of α‐GeTe short‐ranged effects are more evident than in β‐GeTe. This outcome already suggests certain differences in the bonding mechanisms for these materials, in harmony with recent research^[^ ^18^ ^]^ showing that the hexagonal low‐temperature phases are in close bonding proximity to conventional covalent materials, consistently separate from the metastable cubic structures.

### 3.5 What Projected Force Constants Reveal About the Bonding Mechanism

Considering the multitude of proposed bonding mechanisms for these materials, a moment of reflection is needed. When the first signs of such force‐constant maxima were discovered,^[^ ^22^ ^]^ a chain polarization effect along the p‐orbitals was considered responsible for their occurrence. Subsequently, a “hyperbonding” mechanism was proposed which is in line with our observations for α‐GeTe. Upon reviewing our results for β‐GeTe, however, we find that the delocalized interactions might go even further than the proposed three centers.^[^ ^20^ ^]^ As is obvious from Figures 2, 3, 4, all investigated [NaCl]‐type IV–VI materials exhibit long‐range interactions that exist beyond three participating atoms. Additionally, while adjacent Ge vacancies would indeed, as pointed out in the proposed hyperbonding mechanism, distort the local geometry enough to strengthen a 3c‐4e (three‐center four‐electron) bond, the projected force constants in Figure 5 indicate the opposite. The vacancy mostly weakens the three‐center Te–Ge–Te interactions, and the Te–Ge–Te unit surrounded by two vacancies shows the strongest decrease. This is a clear hint toward the participation of many more interacting atoms providing the electron density required for the hyperbonding mechanism. This supports the notion that the entirety of the linear chain is significant for the bond stability, and this very idea is reinforced further by the observations made for α‐GeTe because here the presence of coexisting long‐range and short‐range interactions indicate how the metastable rock‐salt structure may be formed.

**Figure 8:**
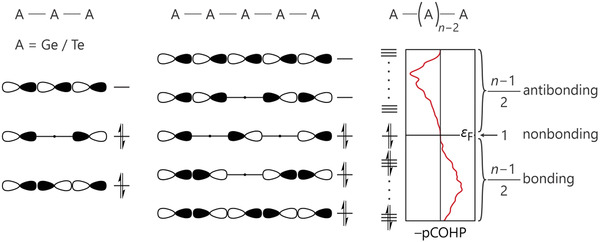
p‐Orbital diagram for an *n*‐center bond, redrawn in the spirit of ref. ^[^ ^32^ ^]^, and its corresponding projected COHP diagram. The proposed literature model agrees well with the projection for σ‐type p‐orbital interaction in the Ge—Te bond of β‐GeTe.

And yet, “hyperbonding” points into the right direction, at least from our point of view. There is an orbital‐based model originally proposed^[^ ^36^ ^]^ for p‐orbital mediated *n*‐center hyperbonding in solids, and it is applicable to the cubic structures. This model represents an extension of the well‐known multicenter bonding concept in the framework of translationally invariant solids. The underlying interactions of *n* p‐orbitals in a one‐dimensional chain composed of species A (either Ge or Te) are depicted in **Figure** **8**; note that, because there is hardly a charge transfer between Ge and Te, substituting A with Ge and/or Te hardly makes any difference. As there is an energetic advantage for such long‐range *n*‐center interactions, this scenario provides a reasonable explanation of the unusual formation of the rock‐salt type in IV–VI materials. Namely, the rock‐salt type is uniquely suited to facilitate *n*‐center bonds along each Cartesian axis due to its rectangularity and, hence, the existence of alternating linear chains of infinite length in all three spatial dimensions. This allows the p‐orbital chains shown in Figure 8 to form *n*‐center bonds along *x*, *y*, and *z*, and such bonding picture covers all the advantages of hyperbonding demonstrated by previous research,^[^ ^20^ ^]^ whilst still being in line with the observations demonstrated in this work. The concept of long‐range multicenter interactions may also account for the bond‐breaking characteristics termed under “metavalency”, which has been proposed to exist between localized and delocalized bonding regimes. Nonetheless, the same characteristics dubbed “metavalent” also arise, naturally so, from the aforementioned multicenter theory without additional assumptions because the bonding valence electrons are delocalized across *n* centers. The recent development of a new orbital‐based descriptor method capable of modelling multi‐center bonding promises to further corroborate this bonding concept.^[^ ^36^ ^]^

Finally, the study of atomic displacement in β‐GeTe also supports the interpretation of long‐range multi‐centered interactions. The distinct shifts in electronic charge and ICOHP induced by disturbing the highly ordered rock‐salt structure of β‐GeTe indicates a clear incentive for dimerization and eventual Peierls distortion. The ability of the rock‐salt type to facilitate orthogonal simultaneous *n*‐center interactions allows the phase to withstand the Peierls instability that leads to the more stable layered α‐GeTe. As such they are a feasible explanation for β‐GeTe's metastability and, consequently, for its unique phase‐change properties.

## 4 Conclusions

Projected force constants from first principles give easy access to both short‐ and long‐range solid‐state atomic interactions that are difficult to isolate by experimental means. The course of the projected forces constants as a function of interatomic distance allows us to characterize and analyze the unusual bonding in phase‐change materials, in particular IV–VI phases. For rock‐salt‐type IV–VI functional materials, the combination of both electronic‐structure chemical‐bonding data by means of COHP and the strong long‐range interactions along linear 1D‐chains found by force‐constant projection suggests germanium‐mediated *n*‐center bonding. This model is well suited to explain the properties commonly found in these materials, such as their unique phase‐change behavior and the recently discovered bond‐breaking mechanism. At the same time, the fascinating physical properties appear as a natural consequence of multicenter bonding, an established molecular quantum‐chemical term, but now explicitly in the context of crystalline matter.^[^ ^37^ ^]^ In addition, it seems advantageous to use the projected force constants as yet another descriptor for tailoring the bonding nature, for example as part of future materials‐design efforts.

## Conflict of Interest

The authors declare no conflict of interest.

## Data Availability

The data that support the findings of this study are available from the corresponding author upon reasonable request.
